# Sero-Epidemiological Survey of Orthopoxvirus in Stray Cats and in Different Domestic, Wild and Exotic Animal Species of Central Italy

**DOI:** 10.3390/v13102105

**Published:** 2021-10-19

**Authors:** Francesca Rosone, Marcello Giovanni Sala, Giusy Cardeti, Pasquale Rombolà, Marina Cittadini, Azzurra Carnio, Roberta Giordani, Maria Teresa Scicluna

**Affiliations:** Istituto Zooprofilattico Sperimentale del Lazio e della Toscana “M. Aleandri”, Via Appia Nuova, 1411, 00178 Rome, Italy; marcello.sala@izslt.it (M.G.S.); giusy.cardeti@izslt.it (G.C.); pasquale.rombola@izslt.it (P.R.); marina.cittadini@izslt.it (M.C.); azzurra.carnio-esterno@izslt.it (A.C.); roberta.giordani@izslt.it (R.G.); teresa.scicluna@izslt.it (M.T.S.)

**Keywords:** orthopoxvirus, cowpox virus, target population, cat, mammals, seroprevalence, zoo

## Abstract

Orthpoxvirus infection can spread more easily in a population with a waning immunity with the subsequent emergence/re-emergence of the viruses pertaining to this genus. In the last two decades, several cases of Orthopoxvirus, and in particular Cowpoxvirus infections in humans were reported in different parts of the world, possibly due to the suspension of smallpox vaccinations. To date, in Italy, few investigations were conducted on the presence of these infections, and because of this a serosurvey was carried out to evaluate Cowpoxvirus infection in feline colonies situated in the province of Rome, since these are also susceptible to other zoonotic viruses belonging to Orthopoxvirus, and from which humans may contract the infection. The sample design was set at an expected minimum seroprevalence of 7.5%, a 5% standard error and 95% confidence level. In parallel, a serological investigation was conducted using convenience sampling in domestic, exotic and wild susceptible animals of the Latium and Tuscany Regions, which are areas in the jurisdiction of the Istituto Zooprofilattico Sperimentale del Lazio e della Toscana, coordinating this study. The serological methods employed were indirect immunofluorescence for 36 sera of nonhuman primate and virus neutralization for 1198 sera of different species. All the 1234 sera examined were negative for the presence of antibodies against Cowpoxvirus, indicating its limited circulation in the areas of investigation. The methodology applied for the serosurveillance could be adopted in the case of outbreaks of this infection and for the evaluation of the spread of this infection in the area of interest, to obtain essential information crucial for animal and public health policies according to the One Health concept.

## 1. Introduction

The Cowpoxvirus (CPXV) belongs to the family *Poxviridae*, genus *Orthopoxvirus* (OPV) and is endemic in northern Europe, with the exception of Ireland and western Russia. During the last 20 years, an increasing number of cases were described in animals in central and southern Europe, and CPXV infection is nowadays considered to be the most common OPV infection occurring in Europe [1]. Human infections caused by CPXV are rare, occur via contact with infected animals [2] and are normally self-limiting, except for immunocompromised patients in which, frequently, major clinical manifestations develop.

Wild rodents are reported as the most likely natural reservoir of CPXV [3,4] infecting a broad range of domestic mammals, including dogs, cats, cattle, horses [2], nonhuman primates and several wild animal species such as elephants, llamas, and other exotic animals kept in captivity at European zoos; not excluding that direct transmission can occur among these species [5,6]. 

The epidemiology of CPXV infection in cats and zoo animals presents a seasonal pattern with a higher incidence in late summer and early autumn (August to October) and a lower one in winter (February to March) [2], probably conditioned by the abundance of the reservoir species populations present.

CPXV is increasingly being detected in cats [7] that can develop lesions and shed large amounts of the virus. Infected cats usually display clinical symptoms, even if latent infections cannot be excluded. Cats may contract the virus by feeding on reservoir species such as rats and mice. Cat to cat transmission is possible and occurs by the oro-nasal route [8]. 

In domestic cats, more than 400 cases of CPXV and CPXV-like [1] (from here on named OPV) infections were reported. Cases of infections may not be suspected or diagnosed by veterinarians or owners as the skin lesions caused by OPV are similar to those due to other viral infections (*Herpesvirus, Calicivirus, Poxvirus*), bacteria superinfections (*Staphylococcus Aureus, Nocardia, Actinomyces*), fungi (*Histoplasma, Sporothrix, Cryptococcus*), food allergens and skin cancer [8,9,10,11,12,13,14].

Serosurveys in other parts of Europe carried out to determine the incidence of CPXV infection in cats in England, Norway, Austria, Germany and Finland [1] defined levels of CPXV antibody positivity from 0 to 16%. 

In the last twenty years, various human cases of OPV infection were reported in Italy. Two of them, respectively, occurred in 2005 and 2007, in Friuli Venezia Giulia (north-eastern Italy) where the virus was isolated in veterinarians reporting scratches caused by infected domestic cats. Serological surveys conducted some years later [15] in veterinary clinics of the same area highlighted the spread of the OPV infection among the feline population with subsequent occupational-related infections among veterinary surgeons. Seroprevalence in veterinarians was 33.3% (12/36), while the overall seroprevalence in cats was 19.5% (44/226), greater than that observed in previous studies in other areas of Europe [16]. In 2009, a CPXV strain was isolated in the Latium Region (central Italy) in five llamas bred for amateur purposes, with serological positivity detected in three animal carers [17]. The fourth case occurred in 2015 [18] when an OPV strain (OPV Abatino, Gruber et al., 2019) was isolated in Tonkean macacques kept in captivity in an animal sanctuary in Latium, with the detection of only one case of an asymptomatic human infection that was serologically detected on the basis of a seroconversion in one staff member [19].

In 2018, a fatal case of infection by an OPV strain was reported in a domestic cat from Siena, Tuscany Region where the isolated virus was genetically closely related to the OPV responsible for the outbreak that occurred in the animal sanctuary in Latium [20]. No symptoms were observed in the in-contact humans.

The increasing cases in humans are probably due to the suspension, since 1980 of smallpox vaccination. This could be the result of a subsequent progressive decline of the basal immunity level against Orthopoxviruses, including CPXV, in the human population [21], together with an apparent increase in the number of cases, respectively, in domestic, synanthropic and wild species such as horses [22], dogs [23] and circus and zoo animals, as well as being due to the growing interest in pet rats, which as described previously, are particularly susceptible and also acting as possible reservoirs. In recent years, some zoonotic OPVs are re-emerging, including the Vaccinia virus, the Monkeypox virus and Buffalopox virus, in areas were previously they were not reported, with the notification of an alarming number of cases worldwide [24,25]. Most outbreaks in endemic countries are reported in rural communities such those caused by cowpoxvirus in Europe and Asia [26], and the new orthopoxvirus-related strains in Alaska, the United States [27] and Georgia [28,29].

As mentioned, OPV outbreaks are usually related to rural populations, but the introduction of the virus in more urbanized and populated areas cannot be excluded. In fact, the human population, that could be more susceptible due to the suspension of smallpox vaccination, could contract the infection from wild and domestic animals that act as intermediate hosts and therefore determine the emergence or re-emergence of the OPV. The ability of OPV to overcome species barriers and circulate among domestic, wild and exotic animals, both in captivity and in the wild, as well as the severe forms in immunocompromised persons, calls for the need to carry out studies targeted on virus spread and its impact on animal and public health [30].

For these reasons, and given the few epidemiological investigations carried out in Italy, the present paper describes a cross-sectional survey aimed at estimating the prevalence of OPV antibodies in a representative sample of stray cats living in registered feline colonies of a Local Health Unit (LHU) (ASL Rome 3) in the Rome metropolis.

In addition, considering the number of species susceptible to the infection, a convenience sample including sera received for other diagnostic investigations were also analysed to estimate the OPV seroprevalence in domestic and wild, allochthonous and/or synanthropic species and exotic animals residing in the Latium and Tuscany Regions, located in Central Italy.

## 2. Biosafety

According to the Italian legislation, Cowpox virus is an etiologic agent belonging to risk class 2 as indicated in Annex 46 of the Legislative Decree 81/2008 and subsequent amendments, and for this, the samples were analysed adopting all the necessary biosecurity measures listed as following:

- directly or indirectly informing the laboratory personnel about the risk of exposure;
- performing tests within a class II vertical laminar flow hood;
- use of personal protective equipment and its proper disposal;
- appropriate disinfection of all contaminated materials and/or sterilization;
- proper handling of OPV suspect biological samples only by personnel vaccinated against Variola virus (Smallpox virus).

## 3. Materials and Methods

### 3.1. First Study: Cross Sectional Survey in Feral Cats

#### 3.1.1. Study Area

The study area of the Rome3 LHU consists of the Fiumicino Municipality and the southwest part of the Rome Municipality (three districts). The latter part covers an area of about 23% of the entire surface of the Rome Municipality and hosts a human population (Istat: National Institute of Statistics data as of 1 January 2020) [31] of approximately 19% of the entire population residing in the same municipality.

**Figure 1:**
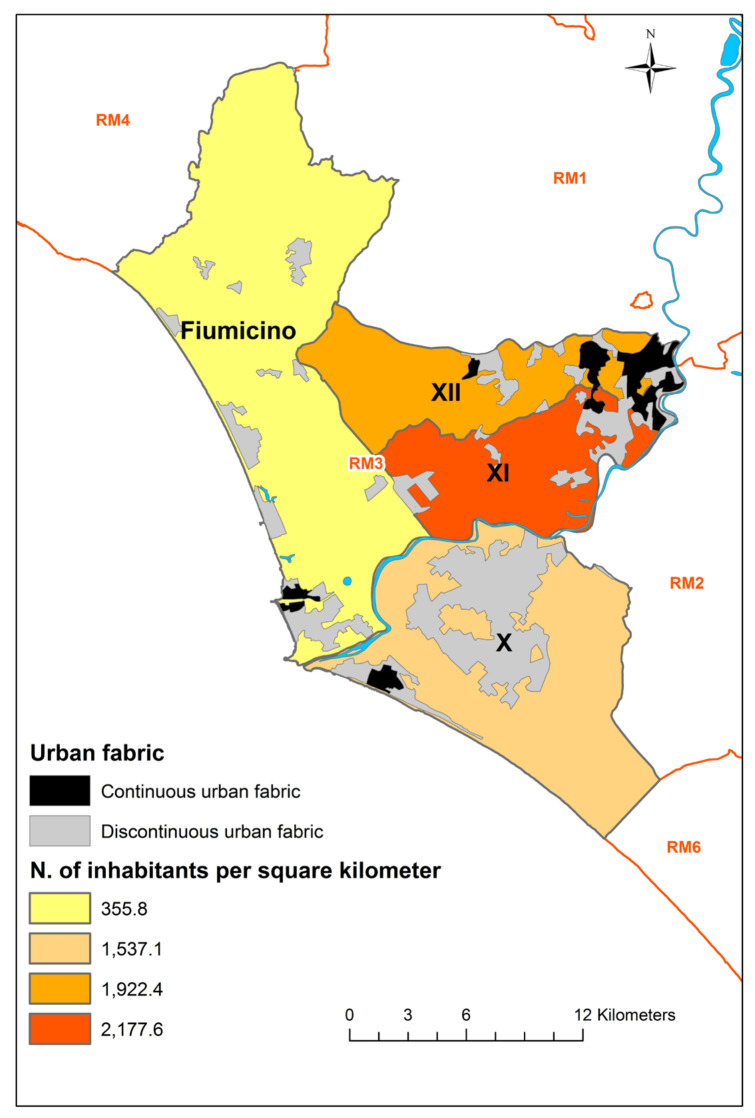
Human population density and urban fabric of the study area (RM followed by a number refers to the LHU of Rome). Layers sources: Corine Land Cover (CLC) 2018 (https://land.copernicus.eu/pan-european/corine-land-cover/clc2018?tab=download, accessed on 25 August 2021); Rome districts (http://websit.cittametropolitanaroma.it/Download.aspx, accessed on 25 August 2021); Italian municipalities (https://www.istat.it/it/archivio/222527, accessed on 25 August 2021); Resident population data sources: http://dati.istat.it/Index.aspx?DataSetCode=DCIS_POPRES1#, accessed on 25 August 2021 (Fiumicino municipality), https://www.comune.roma.it/web/it/i-numeri-di-roma-capitale.page, accessed on 25 August 2021 (Districts X, XI and XII).

In the two coastal areas (Fiumicino District and X District), the urban fabric is mainly discontinuous and concentrated along the coast of the Fiumicino Municipality, and rapidly increases towards the centre of Rome. Figure 1 represents the study area and the boundaries of the four districts, the urban fabric and the human population density. The map was created using ArcGIS 10.8 software.

#### 3.1.2. Population under Study

The data on the cat population were provided by the LHU Roma 3, which detains, as required by law [32,33], the official registry of feline colonies present in the four districts into which it is divided, reporting a total study population of 16,763 cats living in 1466 different feline colonies. The study population was considered as homogeneous for the potential risk of contracting CPXV infection all over the study area as it consists of semidomestic cats living in an urban, semirural environment. Furthermore, all feline colonies are under official health control and are managed on voluntary basis by citizens accredited by the local health authority. All the colonies are monitored for their welfare, and food is regularly provided by the accredited volunteers.

The study aim was to estimate the prevalence of CPXV seropositive cats in the study population with the sample size determined separately for each district, setting an expected minimum seroprevalence of 7.5%, with a 5% standard error and 95% confidence level. These parameters were chosen on what is reported about CPXV circulation in Italy in the limited number of papers published to date.

Given these assumptions, a minimum sample of 107 cats was calculated for each of the four districts of the LHU Roma 3, for a total of 428 samples.

The cats on reception were progressively enrolled in the study at the LHU Roma 3 veterinary clinics during the 2018 sterilization campaign, until the required sample size was reached. Furthermore, each blood sample was accompanied by a medical history sheet of the cat, including the identification of the environmental and behavioural risk factors as described in the section below.

#### 3.1.3. Descriptive and Statistical Analysis

Descriptive analysis of the population under study was conducted and frequency tables were produced. Statistical differences were verified in the distribution for district, age, sex and living environment/area of the samples analysed. The Kruskal-Wallis test was applied for continuous variables with nonparametric distributions, and the Chi Square test with post hoc Bonferroni analysis for categorical data. For both tests, Alpha (significance) was set at 0.05. Analyses were performed using R statistical software (R Core Team, 2020, Austria, version 4.0.3).

### 3.2. Second Study: Convenience Sampling of Other Animal Species

From 2015 to 2018, 799 sera collected from various animal species in several municipalities of Lazio and Tuscany Regions were analysed for the presence of OPV antibodies.

Convenience unrepresentative samples recruited from those submitted for laboratory analyses of other infectious diseases were also included to increase the sample size of our study. The samples consisted of: 60 horse blood sera collected from the equine infectious anaemia surveillance plan; 102 dog sera and 170 cat sera submitted for the verification of antibodies following rabies vaccination, and in case of suspected *Ehrlichia canis*, *Leishmania infantum*, *Coronavirus, Herpesvirus, and Rickettsia conorii* infection; 91 bovine sera collected in 2013 for antibody analysis for *Schmallenberg virus* in twelve farms in Tuscany; 90 sheep sera from 10 farms in Tuscany and one in Lazio, and 30 goat sera from one farm in Tuscany and one in Lazio, collected in 2013 for serological diagnosis of *Blue Tongue* and *Schmallenberg viruses* in the two species (Table 5). In addition, sera were recruited from other species such as fox, roe deer, wolf, wild boar and kangaroos, as reported in Table 5.

Furthermore, 191 sera of different mammals, including nonhuman primates, hosted at the Bioparco in Rome were examined. (Table 5).

All collected sera were stored at −20 °C until analysis.

### 3.3. Serologic Tests

The methods employed in both studies for the detection of OPV antibodies were the indirect immunofluorescence [4] and virus neutralization, based on reports in the literature after carrying out an extensive bibliographic research.

### 3.4. Indirect Immunofluorescence Assay (IFA)

IFA was employed to analyse cat, nonhuman primates (NHP) and small rodent sera.

The samples were tested in two-fold dilutions to define the highest positive titre for the presence of IgM and IgG anti -OPV antibodies, starting from a dilution of 1/20 to 1/640, in phosphate buffered saline (PBS) with a preliminary inactivation of the undiluted serum at 56 °C for 30 min.

Slides for IFA were prepared using Vero E6 cells infected with VACV (Lancy-Vaxina strain, Berna Biotech Ltd., Berna, Swiss).

The cells were harvested when the virus cytopathic effect (CPE) was less than 20–30% (generally around 24 h from the inoculum with a multiplicity of infection of 0.1) and a defined cell concentration was spotted on the slides, which were then air dried, fixed in cold acetone and stored at −80 °C until their use. 

Secondary species-specific (anti-monkey, anti-human, anti-rat and anti-cat) anti-IgM and anti-IgG antibodies conjugated with fluorescein isothiocyanate were added at the dilution indicated by the manufacturer (Sigma-Aldrich, Inc., Burlington, MA, USA). Evans Blue was added as a contrast dye at 1%, directly diluted in the secondary antibody solution. To assess test validity, both negative and positive controls were included, with the latter characterized by the presence of green and bright small fluorescent granules in the cell cytoplasm. 

In case of aspecific fluorescence, samples were examined using different buffers: nonfat dry milk (Bio-rad Lab S.R.L.) at 5%, Tween (Fisher BioReagents) at 0.05%, goat serum (SIGMA-Aldrich) at 1%, foetal bovine serum (FBS—GIBCO) at 10%, to saturate aspecific binding sites.

### 3.5. Virusneutralization Test (VN)

The presence of OPV-specific antibodies was assessed using a readapted version of the VN indicated for the Camelpox virus in the OIE Manual of Diagnostic Tests and Vaccines for Terrestrial Animals 2019 (Chapter 3.9.2, version adopted in May 2014). The VERO cell line was chosen among the several (VERO, BHK 21, MS monkey kidney and BVK168) permissive to OPV replication. In detail, to ensure the presence of a confluent monolayer within 24 h from splitting, a suspension of VERO cells in E-MEM with 10% FBS at a concentration approximately of 3 × 10^5^ cells / ml was used. 

The vaccinia virus (VACV, Lancy-Vaxina strain, Berna Biotech Ltd., Berna, Swiss) was used as antigen. For the VN, 2-fold dilutions of heat-inactivated serum as described above, starting from a dilution of 1/2, were mixed with 100 tissue culture infective doses of virus in 96-well microtiter plates to obtain a final dilution of 1/4. Following incubation at 37 °C for 60 min, VERO cells at the concentration reported above were added to each well.

The plates were read microscopically for viral CPE after 3 days of incubation at 37 °C in a humidified atmosphere at 5% CO_2_. A VN antibody titre ≥1/4 was considered positive. CPE was characterized by ballooning, presence of giant cells and polynuclear syncytia with degeneration and lysis of the monolayer.

### 3.6. Positive and Negative Controls Used in the Serological Asssays

For both the IFA and the VN, the positive control employed was a nonhuman primate serum obtained from the outbreak described by Cardeti et al. [20] provided by the Istituto Nazionale Malattie Infettive Lazzaro Spallanzani, Rome, Italy, while the negative control was a bovine fetal serum after obtaining a negative result in both tests when examing it in thirty replicates. 

## 4. Results

### 4.1. Cross Sectional Survey in Feral Cats

Of the expected 428 samples to be analysed, the total collected was 435, with the following repartition: 114 from District Fiumicino, 115 from District X, 106 from District XI and 100 from District XII. Table 1 reports the census estimates of the feline population under study and the number of sampled animals per district. Data on the distribution of sampled cats according to sex, age and living area are reported in Table 2.

Only animals for which information on both sex and age (*n* = 405) were available were enrolled in the descriptive and statistical analyses: 100 (87.72%) for District Fiumicino, 113 (98.26%) for District X, 95 (89.62%) for District XI and 97 (97%) for District XII. The distribution of females and males for each district was respectively, 57 (57%) and 43 (43%) for District Fiumicino, 74 (65%) and 39 (35%) for District X, 55 (58%) and 40 (42%) for District XI, 67 (69%) and 30 (31%) for District XII. The mean age calculated using the data of the four districts was 22.5 months.

The study area is predominantly represented by urban and rural environment, and for each district the distribution of sampled cats was respectively, 33 (33%) and 45 (45%) for District Fiumicino, 80 (71%) and 32 (28%) for District X, 45 (47%) and 32 (34%) for District XI, and 48 (49.5%) and 48 (49.5%) for District XII. The remaining study area was classified as coastal and others, with few overall numbers of sampled animals collected here; four (1%) for the coastal area and 16 (4%) for the other type of environments. Due to their low number, the samples relating to these last two categories were excluded from the statistical analysis. As reported in the medical history sheet, 37.5% were in possible contact with mice, while for 7.2% no contact was reported. No data about mice contacts were available for the 55.3% of the cats (“unknown” and missing data). Possible contacts with other species (dogs, cats, rodents, foxes, chicken, goats and hedgehogs) were reported for 24% of the sample population.

During the clinical visit carried out in concomitance of the sample collection, only three of the sampled 435 subjects had suspect lesions potentially referable to OPV but without virological and/or serological positivity.

#### 4.1.1. Statistical Analyses Results

No statistically significant differences were observed among the four districts for sex and age, while a significant difference was observed related to the “living area” (*p*= 0.0004). A higher proportion of cats living in urban area in District X (88%) was present with respect to the other districts (33–49.5%).

Given the substantial absence of differences between the four districts for the demographic variables and the considered risk factors, samples collected in the different municipalities were considered as belonging to one study population.

#### 4.1.2. Serological Analyses

Cat sera were not analysed by IFA due to the presence of aspecific fluorescence, which was observed up to a dilution of even 1/320 in the untreated sera and which persisted even when the sera were treated with different protocols considered as efficient for the removal of this problem. In view of this, to avoid loss of sensitivity and specificity, all cat sera were examined in the OPV VN and none of the samples presented specific antibodies. Given these results, and assuming 100% sensitivity and specificity of the assay employed, the theoretical maximum prevalence of seropositive animals in the feline population living in each district of LHU Rome3 ranged between 2.52% (District Fiumicino) to 2.90% (District XII). (Table 3).

Convenience sampling: results of descriptive and serological analyses.

NHP and small rodent sera were examined using IFA without encountering aspecific fluorescence, with all samples resulting negative (Table 4), as were also all negative the sera of the other animal species (Table 5) analysed in the VN for Orthopoxvirus.

However, 78 of 1234 sera, not related to a particular species, presented hemolysis or cytotoxic effects, and for these the sera were diluted and examined beyond the adverse effect to verify if they were positive in the higher dilutions., clear of these alterations.

## 5. Discussion

The zoonotic potential of CPXV infection and its capacity to cause infection in a vast range of mammal species are well established, but the majority of the reported cases was sporadic, making its real prevalence difficult to establish. This is the first serological investigation conducted in the Lazio and Tuscany regions involving colony cats and a wide range of mammalians collected over a period from 2015 to 2018. Two different studies were conducted, the first aiming to detect virus circulation and prevalence in urban feral cats, and the second to identify potential mammal hosts and habitats that could represent a risk for human exposure.

In the present work, the serological results obtained for both feral cats and other mammals were negative, indicating at least the sporadicity of CPXV infection, which, if present, had a low prevalence.

We estimated the maximum prevalence of seropositive animals in the feral cat population living in each district of LHU Rome 3, ranging between 2.52% to 2.90%, which is the result of a theoretical calculation and subject to error, and whose amplitude is dependent on different variables. A greater number of animals sampled would be necessary to calculate the real prevalence of seropositive cats in the target population, to identify the few positive subjects present, considering that real prevalence in the examined population was possibly much lower than the threshold of 7.5% (ER 5%) assumed in the study sampling design.

Randomization flaws could have mitigated the risk of exposure with an underestimation of the seroprevalence in the target population due to the following reasons.

First, the feline colonies enrolled in the study consisted of semidomestic cats regularly fed by volunteers, thus decreasing the probability of encountering the virus reservoir species.

Second, since the sampled cats were those submitted for sterilization, in the majority of cases these were generally under the age of 12 months, thus reducing the probability of having already had an exposure.

Finally, another factor that could have determined the seronegativity is that in the identified sampling area no outbreaks of OPV were ever diagnosed in the past, confirming the limited circulation of the virus and subsequent exposure. On the contrary, it is likely that this risk is higher in stray cats of nonregistered colonies, even present in the same territory, that are more prone to coming into contact with the virus source.

Furthermore, a low number of subjects with suspicious lesions attributable to OPV infection was reported at the time of the clinical visit conducted to collect the blood samples. One explanation of such a low number of suspect cases could be that skin lesions caused by OPV infection often recover completely without being detected, or other aetiology being suspected, and that cat-to-cat transmission in stray cats is rare [7].

Another element to consider is that the effective analytical sensitivity of the VN is less than 100%. Therefore, it is likely that false negatives were present in the samples analysed with a further underestimation of the seroprevalence observed. The probability of this false negativity could have been increased by the low actual prevalence of infection in the population.

Serological negativity could also be correlated with the type of antibodies detected by the technique used. The VN detects the presence of neutralizing antibodies involved in a viral infection. Furthermore, it must be considered that following OPV infection, the persistence of neutralizing antibodies, as well as that of IgM, appear to be of short duration and at low titres [34].

A problem observed during the VN was the presence of a haemolytic and cytotoxic effect of various sera on the cell culture employed, which could have interfered with the method used and that was resolved as described above. Another critical point was that despite the various treatments carried out to reduce or eliminate aspecificity, IFA was not successful for cat sera. Therefore, the VN was finally employed even in view of a probable reduction in the diagnostic sensitivity. In spite of such limitations, this survey provides evidence of a low probability OPV endemicity (CPXV and CPXV-like strains) infection in the population of feline colonies in the areas examined, and furthermore that the infection, even if present, is sporadic and with a low seroprevalence.

To extend the population of investigation, sera from other animal species collected from sick subjects, subjects that died suddenly, or during analysis for surveillance plans and diagnostic purposes of animal infectious diseases, were included in this study. The sporadic nature of OPV infection in such species as reported in the literature further justifies the absence of antibodies in our convenience sample.

Relative to the exotic animals included in our investigations, in most cases they were present in zoos, sanctuaries and/or kept as pets, where rodent control systems should be constantly used, minimizing the possibility of infection.

## 6. Conclusions

From the serological investigations conducted in cats and other mammals, CPXV and CPXV-like infections appeared to be sporadic in both studies as also supported by the literature. From the results of the serological survey (Study I) in colony cats, the estimated maximum serological prevalence suggests a rare occurrence of CPX infection in the study area during the observation period (less than 3%). A possible underestimation of the spread of the infection in cats could be primarily correlated to two limits that emerged from the present study: the sensitivity of the serological techniques used, and the characteristics of the feline colonies enrolled.

Given the geographical conformation of the Italian territory, for many years it was thought that the occurrence of OPV infections could be limited only to sporadic imported cases. On the contrary, the episodes that occurred in the last 15 years justify further investigations on the effective spread of this infection, also in view of the clinical evolution of the disease characterized by lesions that can be easily mistaken with those from other pathogens, and which often recover completely and spontaneously. New cases of OPV with zoonotic potential are being increasingly reported in the world.

OPV infection should be taken in account during the differential diagnosis of skin lesions in cats and domestic animals, including pet rats, particularly when resistant to the treatment of symptomatic therapies. Although CPXV infection is not endemic in cattle or cats, reports of infections transmitted to humans by these species, and also by pet rats and captive exotic animals, have become more frequent [35]. More studies are required to better comprehend the spread of CPXV and other OPV infections, because it was observed that high morbidity and mortality could occur in highly susceptible animal species [23,24,25] and in humans, in particular, due to smallpox [36].

## Figures and Tables

**Table 1:** Census of feline population under study and cats sampled per district.

District	N. Surveyed Cats	N. Surveyed Colonies	N. Cats to Be Sampled (N. Colonies)	N. Sampled Cats
District Fiumicino	3645	302	107 (9)	114
District X	8124	714	107 (9)	115
District XI	2924	265	107 (10)	106
District XII	2070	185	107 (10)	100
TOTAL	16763	1466	428	435

**Table 2:** Distribution of all sampled (*n* = 435) cats per district according to sex, age and living area.

District	Sex (M/F) *n* = 429	Age (≤1; >1 Year) *n* = 407	Cats Living in Rural/Urban Area *n* = 387
District Fiumicino	52/58	75/27	49/40
District X	40/75	95/18	33/81
District XI	43/61	69/26	35/50
District XII	31/69	70/27	48/51
Total	166/263	309/98	165/222

**Table 3:** Estimation of the theoretical maximum prevalence for each district given the negative result of all samples and considering sensitivity and specificity of the SN equal to 100%.

Area/District	Registered Cats	Surveyed Colonies	Examined Cats	Theoretical Maximum *p*% (Se—Sp 100%)	Estimate of the Maximum Number of Positive Cats in the Population
Fiumicino	3645	302	114	2.52%	92
District X	8124	714	115	2.56%	208
District XI	2924	265	106	2.67%	78
District XII	2070	185	100	2.90%	60

**Table 4:** Sera analysed by IFA.

Animals Living at Bioparco		Animals Living in Other Areas *	
Species	Examined	Species	Examined
JAPANESE MACAQUE *(Macaca fuscata)*	1	CAPIBARA *(Hydrochoerus hydrochaeris)*	2
BARBARY MACAQUE *(Macaca sylvanus)*	1	RING-TAILED LEMUR *(Lemur catta)*	2
NORTHERN PIG-TILED MACAQUE *(Macaca leonina)*	1	JAPANESE MACAQUE *(Macaca fuscata)*	2
CRAB-EATING MACAQUE *(Macaca fascicularis)*	24	MANDRILL *(Mandrillus sphinx)*	2
CHLOROCEBUS *(Chlorocebus spp.)*	1	TOTAL	8
TOTAL	28		

* farm, recovery center, in the wild.

**Table 5:** Sera analysed by VN.

Animals Living at Bioparco		Animals Living in Other Areas *	
Species	Examined	Species	Examined
COMMON ELAND *(Taurotragus oryx)*	7	CAT (*Felis silvestris*)	170
ADDAX *(Addax nasomaculatus)*	1	DOG (*Canis lupus familiaris*)	102
BLUEBUCK *(Hippotragus leucophaeus)*	1	GUINEA PIG (*Cavia porcellus*)	1
BLACKBUCK *(Antilope cervicapra)*	1	HORSE (*Equus caballus*)	60
DONKEY *(Equus asinus)*	1	CATTLE (*Bos taurus*)	91
BANTENG *(Bos javanicus)*	1	SHEEP (*Ovis aries*)	90
EUROPEAN BISON *(Bison bonasus)*	1	GOAT (*Capra hircus*)	30
BACTRIAN CAMEL *(Camelus bactrianus)*	5	ROE DEER (*Capreolus capreolus*)	1
KANGAROO *(Macropus giganteus)*	1	FOX (*Vulpe vulpes*)	3
CAPYBARA *(Hydrochoerus hydrochaeris)*	5	WOLF (*Canis lupus*)	2
DOMESTIC GOAT *(Capra hircus)*	2	WILD BOAR (*Sus scrofa*)	2
CERCOCEBO *(Cercocebus spp.)*	1	BADGER (*Meles meles*)	3
CERCOPITHECO *(Chlorocebus spp.)*	1	HEDGEHOG (*Erinaceus europeus*)	3
SIKA DEER *(Cervus nippon)*	9	WEASEL (*Mustela nivalis*)	1
RED DEER *(Cervus elaphus)*	1	CERCOPITHECO (*Chlorocebus spp*.)	1
WATERBUCK *(Kobus ellipsiprymnus)*	1	RED-HANDED TAMARIN (*Saguinus midas*)	2
FALLOW DEER *(Dama dama)*	15	CERCOCEBO (*Cercocebus spp*.)	1
AFRICAN BUSH ELEPHANT *(Loxodonta africana)*	2	BARBARY MACAQUE (*Macaca sylvanus*)	1
ASIAN ELEPHANT *(Elephas maximus)*	1	CYNOMOLGUS *(Macaca fascicularis)*	2
GREY SEAL *(Halichoerus grypus)*	2	NORTHERN PIG-TAILED MACAQUE (*Macaca leonina*)	1
FERRET *(Mustela putorius furo)*	2	KANGAROO (*Macropus spp.*)	1
DAMA GAZZELLE *(Nanger dama)*	1	LYNX (*Linx linx*)	3
NORTHERN GIRAFFE *(Giraffa camelopardalis)*	2	OCELOT (*Leopardus pardalis*)	1
GUANACO *(Lama guanicoe)*	1	TOTAL	572
RING-TAILED LEMUR *(Lemur catta)*	3		
AFRICAN WILD DOG *(Lycaon pictus)*	1		
NILE LECHWE *(Kobus megaceros)*	68		
JAPANESE MACAQUE *(Macaca fuscata)*	1		
DOMESTIC PIG *(Sus scrofa domesticus)*	1		
VIETNEMENSE PIGLET *(Sus scrofa domesticus)*	1		
MANDRILL *(Mandrillus sphinx)*	2		
INDIAN MUNTJAC *(Muntiacus muntjak)*	2		
PATAGONIAM MARA *(Dolichotis patagonum)*	1		
MUFLON *(Ovis aries musimon)*	28		
BORNEAN ORANGUTAN *(Pongo pygmaeus)*	1		
BROWN BEAR *(Ursus arctos)*	1		
BUSHPIG *(Potamochoerus larvatus)*	1		
BROWN RAT *(Rattus norvegicus)*	1		
BLACK RAT *(Rattus rattus)*	1		
EUROPEAN HEDGEHOG *(Erinaceus europaeus)*	1		
SOUTH AMERICAN TAPIR *(Tapirus terrestris)*	1		
HIMALAYAN TAHR *(Hemitragus jemlahicus)*	3		
MOUNTAIN ZEBRA *(Equus zebra)*	1		
CATTLE *(Bos taurus)*	1		
DOMESTIC YAK *(Bos grunniens)*	6		
TOTAL	191		

* farm, recovery center, in the wild.

## Data Availability

Not applicable.
